# Primary malignant pericardial tumour in Lynch syndrome

**DOI:** 10.1186/s12885-020-6677-y

**Published:** 2020-03-06

**Authors:** Pasquale Paolisso, Giulia Saturi, Alberto Foà, Maristella Saponara, Margherita Nannini, Maria Abbondanza Pantaleo, Ornella Leone, Daniela Turchetti, Daniele Calistri, Carlo Savini, Davide Pacini, Carmine Pizzi, Nazzareno Galiè

**Affiliations:** 1 Department of Experimental, Diagnostic and Specility Medicine - DIMES- Sant’Orsola-Malpighi Hospital, University of Bologna, Bologna, Italy; 2 Department of Pathology, University of Bologna, Azienda Ospedaliera S. Orsola-Malpighi of Bologna, Bologna, Italy; 3 Department of Medical and Surgical Sciences, Center for studies on Hereditary Cancer, University of Bologna, Bologna, Italy; 4 Istituto Scientifico Romagnolo per lo Studio e la Cura dei Tumori (IRST), IRCCS, Meldola, Italy; 5 Cardiac Surgery Unit, Cardio-Thoracic-Vascular Department, S. Orsola Hospital, Alma Mater Studiorum - University of Bologna, Bologna, Italy

**Keywords:** Lynch syndrome, Pericardial tumour, MSH2

## Abstract

**Background:**

This case represents the first report of malignant primary cardiac tumour in a patient with Lynch Syndrome associated with MSH2 pathogenic variant.

**Case presentation:**

A 57-year-old woman with previous ovarian cystadenocarcinoma was admitted to the emergency room for hematic pericardial effusion. Multimodal diagnostic imaging revealed two solid pericardial vascularized masses. After pericardiectomy, the final histological diagnosis was poorly differentiated pleomorphic sarcomatoid carcinoma. During follow-up she developed an ampulla of Vater adenocarcinoma. Genetic analysis identified an MSH2 pathogenic variant.

**Conclusion:**

This case contributes to expand the tumour spectrum of Lynch syndrome, suggesting that MSH2 pathogenic variants cause a more complex multi-tumour cancer syndrome than the classic Lynch Syndrome. In MSH2 variant carriers, symptoms such as dyspnoea and chest discomfort might alert for rare tumours and a focused cardiac evaluation should be considered.

## Background

Lynch syndrome (LS) is an autosomal dominant disorder caused by a germline mutation in one of the DNA mismatch repair (MMR) genes (MLH1, MSH2, MSH6, PMS2) or by a loss of expression of MSH2 due to deletion in the EPCAM gene [1]. It is estimated that 1 in 279 people carry mutations in DNA mismatch repair genes [2]. Patients with MMR gene mutations have a significantly increased risk for cancer [3, 4]; indeed, LS is the most common inherited colorectal susceptibility syndrome, accounting for approximately 3% of newly diagnosed cases of colorectal cancer and 3% of cases of endometrial cancer [5]. Although the predominant malignancies are colorectal and endometrial cancer, a wide variety of malignancies is reported, with sites including ovary, upper urinary tract, stomach, small bowel, pancreas, biliary tract, skin, and brain [6]. However, to the best of our knowledge, no reports have described the occurrence of primary cardiac tumours in LS patients. Herein, we report a case of pleomorphic sarcomatoid pericardial carcinoma in a LS patient.

## Case presentation

A 57-year-old woman was admitted to our emergency room for worsening dyspnoea. Her past medical history included salpingo-ovariectomy for an ovarian cystadenocarcinoma in 1996 (15 years negative oncological follow-up). Unfortunately, histological details of this tumour were unavailable.

**Fig. 1:**
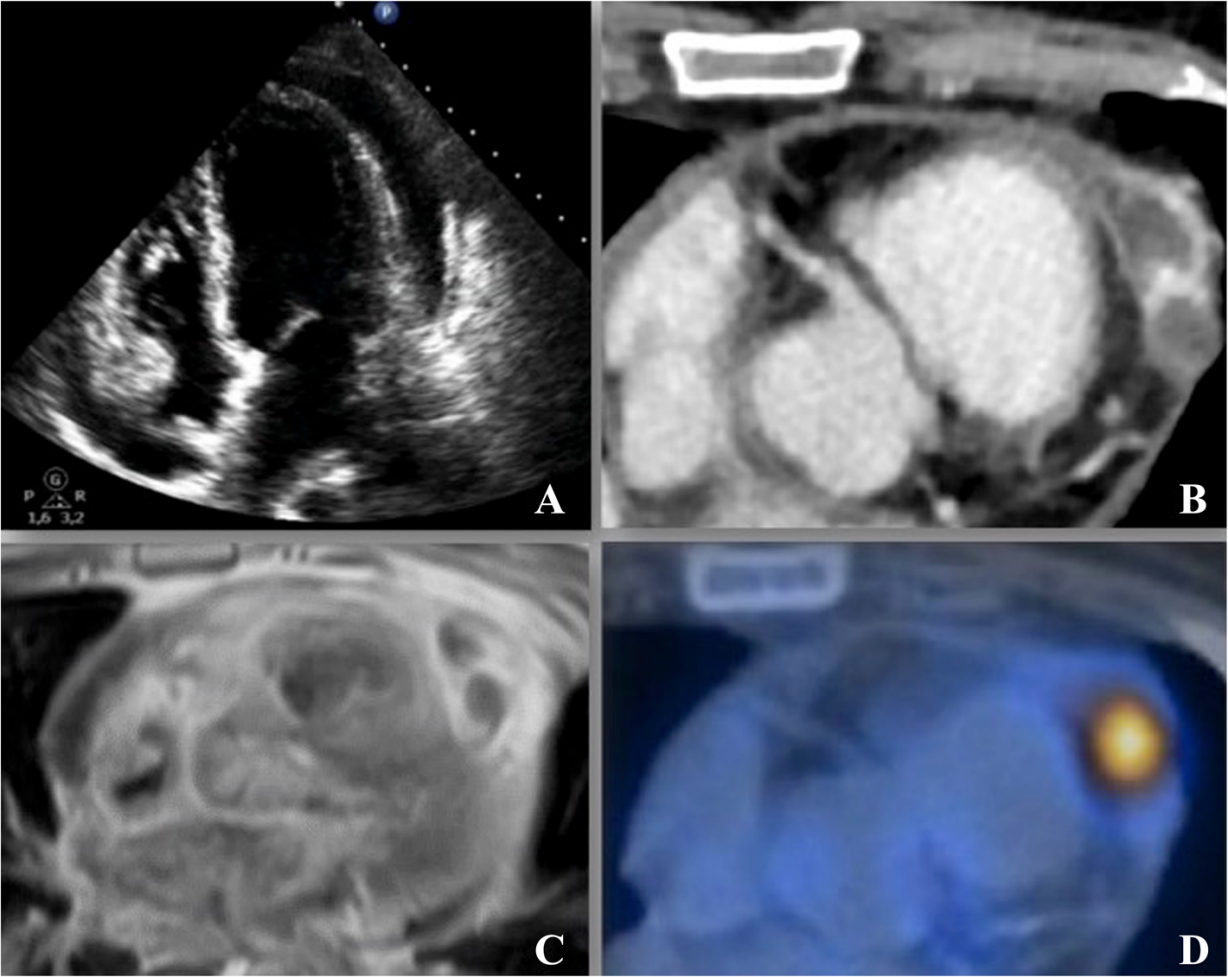
Diagnostic imaging. (A) Transthoracic echocardiograph showing ubiquitous pericardial effusion (max 22 mm) with initial signs of hemodynamic instability. (B) CT scan showing pericardial effusion localized near the right ventricle apex with marked contrast enhancement. (C) Cardiac MR showing two contiguous oval formations, on the anterior pericardial recess, (12 × 15 mm) with fluid/suprafluid content, thin walls and intense contrast impregnation. (D) ^18^F-FDG PET/CT showing a focal area of radiotracer hyperaccumulation at the anterior pericardium recess

At clinical examination the patient appeared sweaty, with low blood pressure and turgor of jugular veins (JVP: 12 cmH2O). Transthoracic echocardiography (Fig. 1a) showed ubiquitous pericardial effusion (max 22 mm) with initial signs of hemodynamic instability; pericardiocentesis was thus performed with aspiration of 400 cc of hematic fluid. The cytological examination for malignant neoplastic cells was negative.

Laboratory findings revealed a normal blood cell count, negative autoimmunity, microbiological and oncologic screening with the exception of CA-125: 247 U/ml (related to pericardial effusion).

Chest-abdomen computed-tomography (CT) (Fig. 1b) revealed a pericardial effusion at the right ventricle apex with focal areas of intense enhancement, compatible with ongoing bleeding. No liver, spleen, pancreas, adrenal and kidney lesions were observed.

Considering the echocardiographic stability of the pericardial effusion, in contrast with the CT findings, a cardiac magnetic-resonance (MR) (Fig. 1c) was performed showing two contiguous oval formations, on the anterior pericardial recess, both 12 × 15 mm with fluid/suprafluid content, thin walls and intense contrast impregnation.

The diagnostic work-up was completed by a 18F-labelled 2-fluoro-2-deoxy-D-glucose Positron-Emission-Tomography/Computed Tomography (^18^F-FDG PET/CT) and a focal area of radiotracer hyper-accumulation in the anterior pericardium recess (SUV max 12.6) was identified (Fig. 1d).

Diagnostic suspicion therefore shifted to a vascularized solid pericardial lesion, and the patient underwent pericardiectomy through a left sub-mammary mini-thoracotomy approach.

**Fig. 2:**
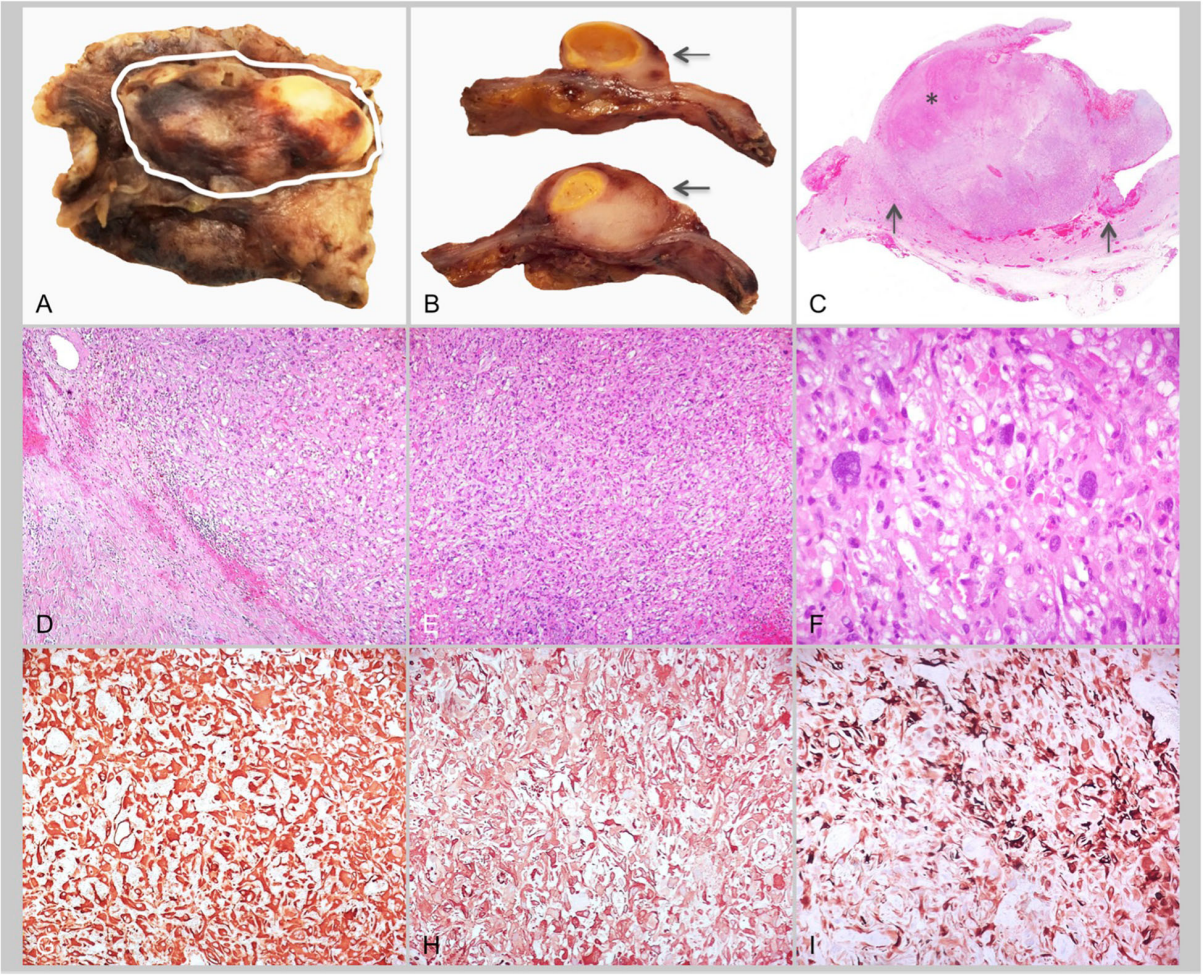
Cardiac mass pathology. Macroscopic appearance of neoplastic mass within the pericardial specimen (A: white circle) and on transverse cuts (B, arrow). The histology macrosection (C) shows infiltration of pericardial tissue by malignant proliferation (arrows) and an extensive area of necrosis (asterisk). At higher magnification, pleomorphic spindle and epithelioid cells are evident (D-E: Haematoxylin-Eosin 100x) as are multinucleated cells (F: Haematoxylin-Eosin 400x). Immunohistochemistry showing diffuse, strong immunostaining for vimentin (G: 200x) and smooth muscle actin (H: 200x) and, in multifocal areas, for cytokeratin-7 (I: 200x)

The surgical specimen was a piece of pericardium with a 4 × 2 cm polylobate mass, constituted by a solid greyish tissue and a necrotic yellowish nodular area (Fig. 2a-c). Histology revealed a malignant proliferation of pleomorphic spindle and epithelioid cells, some multinucleated, growing in solid nests and/or in a storiform pattern. The neoplasia showed atypical mitosis (3/10 mitotic index) and extensive necrotic areas (> 50% of the tumour surface) (Fig. 2d-f). At immunohistochemistry, neoplastic cells were diffusely positive for vimentin and smooth-muscle actin and, in multifocal areas, for cytokeratin7 (Fig. 2g-i). Table 1 summarizes the immunohistochemical results. Despite some atypical aspects of the overall picture, histologic and immunophenotypic findings indicated the final diagnosis of poorly differentiated pleomorphic sarcomatoid carcinoma (pT1Nx M0).

**Table 1:** Immunohistochemistry results

Antibody	Result
Vimentin	diffuse, strong immunostaining
Smooth muscle actin	diffuse, moderate immunostaining
Cytokeratin 7	multifocal, moderate immunostaining
Cytokeratin 5–6	focal, strong immunostaining
Cytokeratin 19	very focal, strong immunostaining
P53	diffuse, strong immunostaining
Myogenin	negative
Desmin	negative
Calretinin	negative
Wide-spectrum cytokeratin	negative
S100 protein	negative
CD31	negative
CD34	negative
Factor VIII	negative
Epithelial membrane antigen	negative
SOX-1	negative
Mart1-MelanA	negative
HMB-45	negative
CD45	negative
Synaptophysin	negative
WT1	negative
PAX 8	negative
KBA	negative
MDM2	negative

Considering the radical nature of the surgery, the absence of further distant disease localizations, the low proliferative index and the lack of scientific evidence that an adjuvant medical treatment reduces the risk of disease recurrence, we started a programme of close clinical-instrumental surveillance.

Cardiac-MR and ^18^F-FDG-PET/CT performed during follow-up revealed no lesions either in the residual pericardium or in the cardiac chambers until June 2018 when the MR showed no relapse of cardiac disease but did reveal a new hepatic lesion. ^18^F-FDG-PET/CT excluded hepatic involvement but identified a focal area of radiotracer hyperaccumulation in the duodenum (SUV max: 14.4). In order to further characterize such lesion, an echoendoscopy was performed showing a 25 mm mass near Vater’s ampulla, infiltrating biliary and pancreatic tracts. Histological analysis indicated an infiltrating adenocarcinoma.

The patient underwent pancreaticoduodenectomy and histological examination confirmed the presence of moderately differentiated ampulla of Vater adenocarcinoma with polypoid extension in the biliary tract and in the duct of Wirsung; there was no involvement of the lymph nodes and the surgical margins were neoplasia-free (pT2N0M0, TNM stage IB).

The patient did not undergo chemotherapy treatment due to the disease staging but continued with close clinical and instrumental follow-up. Chest-abdomen-CT and cardiac-MR performed during follow-up did not show disease relapse either in the cardiac chambers and pericardium or in the duodenum.

**Fig. 3:**
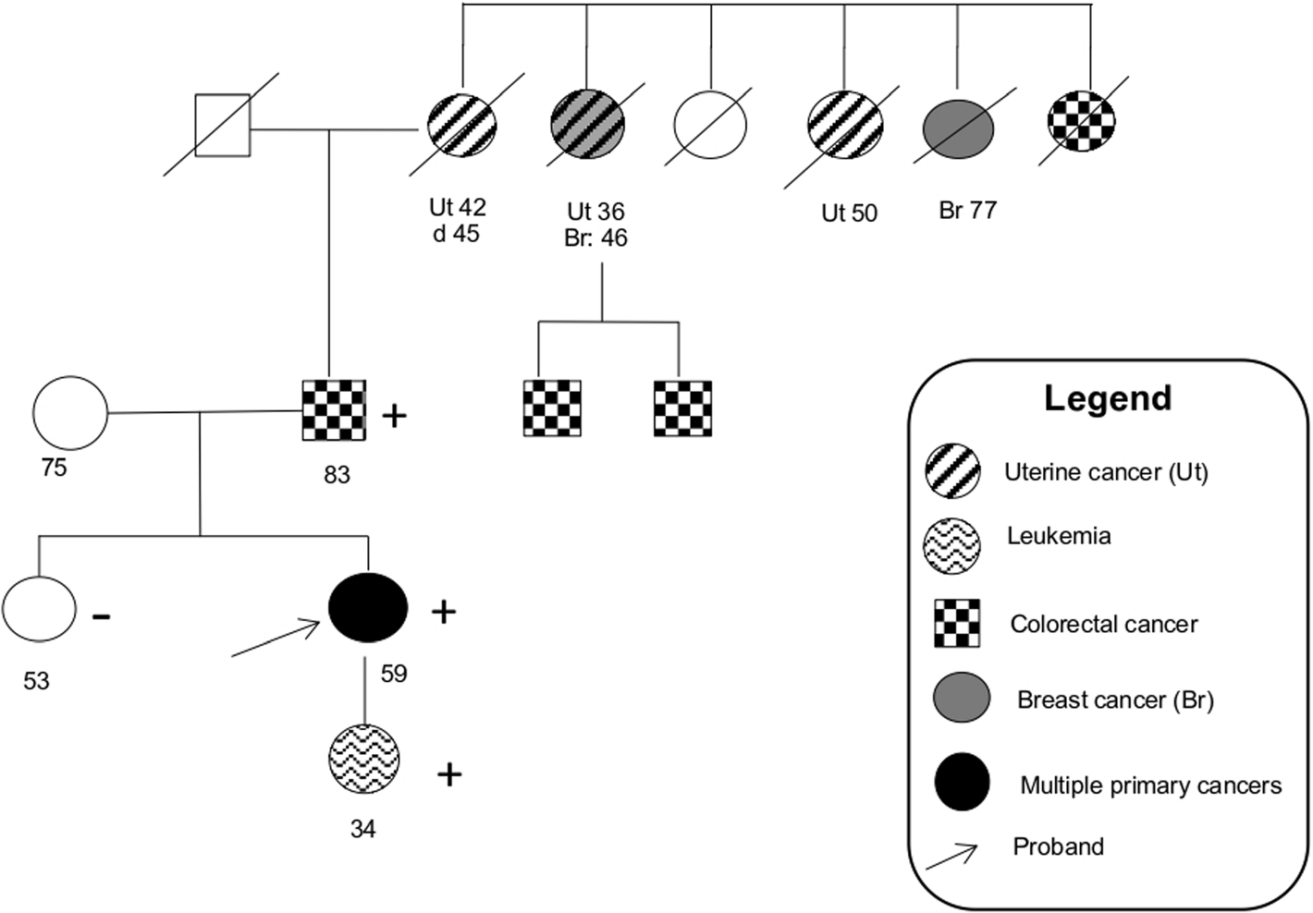
Family. Pedigree. Square symbols represent males, circles females. Clinical status is indicated by open symbols (unaffected) and filled symbols (affected), with filling type indicating cancer diagnosis as detailed in the legend. The presence/absence (+/−) of the gene variant in tested members is shown. Numbers under the symbols show age at diagnosis (after disease abbreviation), age at death (after “d”) or age at pedigree collection (no characters before)

Due to the very rare cardiac tumour histotype, a molecular characterization of the cardiac tumour (other tumours tissues were unavailable) was performed by FOUNDATIONONE®CDx, a commercially available Next Generation Sequencing (NGS)-based analysis of hundreds of cancer-related genes. Pericardial lesion analysis showed mutations in five genes: CD79A (R131fs*61), SETD2 (F1132fs*22), MLH1 (N168fs*4), MSH2 (F58Ilefs*27), TP53 (R175H), as well as a high tumour mutational burden and an intermediate microsatellite status. In the meantime, the patient was referred to our Cancer Genetics Clinic for genetic counselling because of the personal history of early onset multiple malignancies suggesting an inherited cancer predisposition. Extended pedigree collection **(**Fig. 3**)** showed a history of uterine cancer in the paternal grandmother and in two of her sisters, while two paternal cousins were reported to have developed colorectal cancer. Clinical cancer genetic testing (NGS-based 27-gene panel) detected the germline pathogenic variant c.171_172insATCCGGGTGA (p.F58Ilefs*27) of the MSH2 gene, which was diagnostic for LS^7^. This made it possible to implement a proper endoscopic surveillance programme for the patient and to offer predictive genetic testing to relatives. The daughter, who as a child had been treated for Acute Lymphoblastic Leukemia, was proven to carry the MSH2 variant as well. Targeted testing was then offered to other family members and demonstrated that the variant had been inherited from the father, who developed colorectal cancer shortly after the genetic diagnosis in the proband (at the age of 83), while was absent in the healthy 53-year-old proband’s sister. Among the clinically relevant mutations found in the tumour tissue, the MSH2 variant was the same as detected by germline testing, while the MLH1 and TP53 mutations were confirmed to be somatic by both the germline panel test and targeted Sanger sequencing.

## Discussion and conclusion

Primary cardiac tumour are uncommon, with an estimated prevalence around 0.02–0.056%, among these the prevalence of primary pericardial neoplasms ranges from 0.001 to 0.007% [7–9]. This is the first report of malignant primary cardiac tumour in a patient with LS associated with a MSH2 pathogenic variant. Among LS patients, the majority of those developing extraintestinal malignancies are MSH2 mutation carriers [10, 11]. Recent studies have shown that LS patients may present different neoplastic formations according to age, gender and type of MMR defect [3, 4]. Furthermore, a worse prognosis has emerged among the elderly [4].

Our case supports this evidence, since the patient did not develop the predominant Lynch malignancies (colorectal and endometrial cancer), while she did develop rare LS manifestations (ovarian cancer, Vater papilla carcinoma) and the unusual cardiac tumour hereby described. The case was further characterized throughout tumour NGS analysis, leading to the identification of additional somatic events, with a high mutation burden which is consistent with the underlying DNA MMR defect [12]. This comprehensive approach was essential for a proper practical management of our patient. In fact, the extraordinary rarity of the case together with the personal history of cancer raised the suspicion of DNA germline predisposition which ultimately led to genome profiling in order to identify molecular alterations potentially beneficial for diagnosis, treatment and follow-up. MSH2 inactivation leads to MMR defects, MSI, and a high mutational burden, which may have an important implication in predicting response to immunotherapies like pembrolizumab and nivolumab [13, 14] . In case of non-operable disease recurrence or new non-operable cancer, in addition to conventional chemotherapies, the patient could have been a candidate to receive immunotherapy, currently widely used and effective in other solid neoplasms such as melanoma, non-small cell lung cancer and renal cell carcinoma [15]. Our clinical report described a cardiac involvement in a patient with a genetic mutation in MSH2 causative of LS. In this setting, it was crucial to perform endoscopic surveillance of our patient [16] and genetic analysis of her first-degree relatives [17]. This case contributes to expand the tumour spectrum of LS, suggesting that MSH2 pathogenic variants cause a more complex multi-tumour cancer syndrome than the classic form of LS. Therefore, in MSH2 variant carriers, the occurrence of symptoms such as dyspnoea and chest discomfort should advise clinicians to perform a focused cardiac evaluation by 2D-echocardiogram as rare tumours might be detected.

## Data Availability

Not applicable.

## Abbreviations

^18^F-FDG PET/CT: 18F-labelled 2-fluoro-2-deoxy-D-glucose Positron-Emission-Tomography/Computed Tomography
CT: Computed-tomography
LS: Lynch Syndrome
MMR: Mismatch repair
MR: Magnetic-resonance
NGS: Next generation sequencing
